# The effect of inhaled extrafine beclometasone dipropionate/formoterol fumarate/glycopyrronium bromide on distal and central airway indices, assessed using Functional Respiratory Imaging in COPD (DARWiIN)

**DOI:** 10.1186/s12931-023-02549-5

**Published:** 2023-10-06

**Authors:** Gwen S. Skloot, Alessandro Guasconi, Benjamin R. Lavon, George Georges, Wilfried De Backer, Dmitry Galkin, Mauro Cortellini, Ilaria Panni, Jason H. T. Bates

**Affiliations:** 1 Global Clinical Development, Chiesi Farmaceutici SpA, Parma, Italy; 2 FLUIDDA, Kontich, Belgium; 3 Department of Respiratory Medicine, University of Antwerp, Antwerp, Belgium; 4 Departments of Medicine, Larner College of Medicine, University of Vermont, Burlington, VT USA; 5 Chiesi USA, Inc., Cary, NC USA

## Abstract

**Background:**

This study, in patients with symptomatic chronic obstructive pulmonary disease (COPD), explored switching therapy from non-extrafine high-dose inhaled corticosteroid/long-acting β_2_-agonist (ICS/LABA; fluticasone propionate/salmeterol [FP/SLM]) to extrafine medium-dose beclometasone dipropionate/formoterol fumarate dihydrate/glycopyrronium (BDP/FF/G), both via dry-powder inhaler. Functional Respiratory Imaging, a quantitative computed tomography method with 3D reconstructions of pulmonary anatomy, was used to assess airway geometry and lung function.

**Methods:**

Patients receiving a stable ICS/LABA regimen for ≥ 8 weeks were switched to FP/SLM 500/50 µg, one inhalation twice-daily (high-dose ICS) for 6 weeks. After baseline assessments (Visit 2 [V2]), therapy was switched to BDP/FF/G 100/6/10 µg, two inhalations twice-daily (medium-dose ICS) for 6 weeks, followed by V3. The primary endpoints were percentage changes in specific image-based airway volume (siV_aw_) and resistance (siR_aw_) from baseline to predose at V3 (i.e., chronic effects), assessed at total lung capacity (TLC) in central and distal lung regions. Secondary endpoints included siV_aw_ and siR_aw_ changes from pre-dose to post-dose at V2, and from pre-dose to post-dose at V3 at TLC (i.e., acute effects), and chronic and acute changes in siV_aw_ and siR_aw_ at functional residual capacity (FRC). Pre-dose forced expiratory volume in 1 s (FEV_1_) and COPD Assessment Test (CAT) were also assessed.

**Results:**

There were no significant changes in pre-dose siV_aw_ or siR_aw_ at TLC from baseline to V3, although at FRC there was a significant decrease in mean siR_aw_ in the distal airways (− 63.6%; p = 0.0261). In addition, in the distal airways there were significant acute effects at TLC on mean siV_aw_ and siR_aw_ (siV_aw_: 39.8% and 62.6%; siR_aw_: − 51.1% and − 57.2%, V2 and V3, respectively; all p < 0.001) and at FRC at V2 (siV_aw_: 77.9%; siR_aw_: − 67.0%; both p < 0.001). At V3, the mean change in pre-dose FEV_1_ was 62.2 mL (p = 0.0690), and in CAT total score was − 3.30 (p < 0.0001).

**Conclusions:**

In patients with symptomatic COPD receiving high-dose ICS/LABA, adding a long-acting muscarinic antagonist while decreasing the ICS dose by switching to medium-dose extrafine BDP/FF/G was associated with improved airway indices, especially in the distal airways, together with improvements in respiratory health status.

*Trial registration* ClinicalTrials.gov (NCT04876677), first posted 6th May 2021

**Supplementary Information:**

The online version contains supplementary material available at 10.1186/s12931-023-02549-5.

## Background

An extrafine formulation of a single-inhaler triple combination of the inhaled corticosteroid (ICS) beclometasone dipropionate (BDP), the long-acting β_2_-agonist (LABA) formoterol fumarate dihydrate (FF), and the long-acting muscarinic antagonist (LAMA) glycopyrronium (G) is approved for maintenance treatment of chronic obstructive pulmonary disease (COPD) and asthma. Three large, one-year studies evaluated the efficacy and safety of this triple combination in patients with COPD; in all three, BDP/FF/G was administered via a pressurised metered dose inhaler (pMDI). In TRILOGY, BDP/FF/G provided superior bronchodilation to BDP/FF, with a 23% reduction in the rate of moderate-to-severe exacerbations and significant improvements in health status (assessed using St George’s Respiratory Questionnaire) [1]. In TRINITY, BDP/FF/G provided superior bronchodilation, a 20% reduction in the rate of moderate-to-severe exacerbations, and significant improvements in health status compared to tiotropium [2]. Finally, in TRIBUTE, BDP/FF/G reduced the rate of moderate-to-severe exacerbations by 15% compared with the fixed-dose combination of indacaterol and glycopyrronium [3]. More recently, a dry powder inhaler (DPI) extrafine formulation of BDP/FF/G has been developed. In a 28-day crossover study in patients with COPD (TRI-D), BDP/FF/G administered via the DPI demonstrated similar efficacy and safety to BDP/FF/G administered via the pMDI [4].

Dose-related incidences of some ICS adverse effects have been reported (e.g., pneumonia [5]). Thus, there is a need to evaluate the effects of lowering the ICS dose whilst adding a LAMA, especially in patients on a high-strength ICS/LABA combination who switch to triple therapy combinations with a medium-strength ICS component. The current study (DARWiIN) aimed to explore the effect of switching therapy in patients with symptomatic COPD from non-extrafine high-dose ICS/LABA (fluticasone propionate/salmeterol [FP/SLM]) to extrafine medium-dose BDP/FF/G, both administered with a DPI. Airway geometry and lung function were assessed using Functional Respiratory Imaging (FRI), a quantitative computed tomography (CT) method that produces 3D reconstructions of the pulmonary anatomy from which metrics of pulmonary structure and function are calculated, the latter using computational fluid dynamics (CFD).

## Methods

**Fig. 1:**
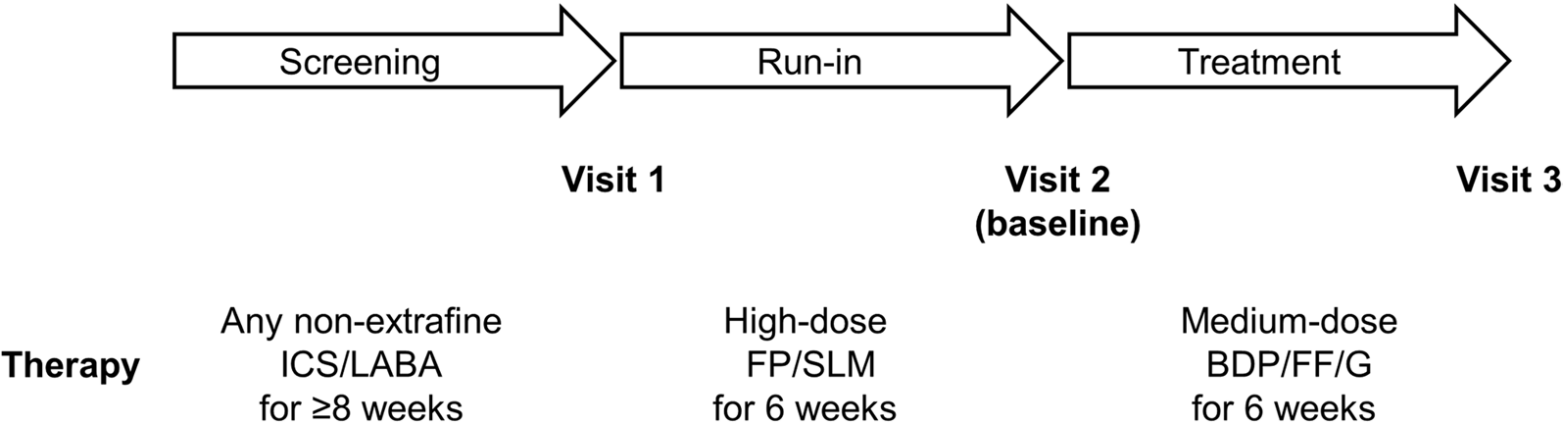
Study schematic. FP/SLM: fluticasone propionate/salmeterol; BDP/FF/G: beclometasone dipropionate/formoterol fumarate/glycopyrronium bromide

This was a multicentre, open-label, single-arm study (Fig. 1). At Visit 1, eligible patients had their COPD maintenance therapy (any non-extrafine ICS/LABA) switched to non-extrafine FP/SLM 500/50 µg DPI, one inhalation twice-daily (i.e., high-strength ICS component) for 6 weeks. Baseline assessments were then performed at Visit 2, before maintenance therapy was switched to extrafine BDP/FF/G 100/6/10 µg DPI, two inhalations twice-daily (i.e., medium-strength ICS component) for 6 weeks, with patients then returning to the study site for Visit 3.

At the screening visit (Visit 1), subjects underwent spirometry (including forced expiratory volume in 1 s [FEV_1_] and forced vital capacity [FVC]) pre- and post-bronchodilator to assess reversibility, with body plethysmography and the COPD Assessment Test (CAT) evaluated pre-dose. At Visits 2 and 3, assessments were repeated, although spirometry was only conducted pre-dose (i.e., before administration of BDP/FF/G). Multidetector computed tomography (MDCT) of the chest was performed at inspiratory breath hold (i.e., total lung capacity [TLC]) and expiratory breath hold (i.e., functional residual capacity [FRC]), pre-dose and 1–2 h post-dose at Visits 2 and 3. The MDCT images were post-processed with FRI to derive specific image-based airway volume (siV_aw_) and resistance (siR_aw_). ‘Specific’ refers to a normalisation procedure to adjust for the influence of inter- and intra-subjective variability in lung volume (see the Additional file 1 for full details). Other MDCT/FRI endpoints included perfusion mapping (as measured by pulmonary vascular volume distribution), lobar volume, and air trapping (see Additional file 1).

Recruited patients were adults ≥ 40 years of age with a diagnosis of COPD (Global Initiative for Chronic Obstructive Lung Disease 2020 criteria [6]). Other inclusion criteria included post-bronchodilator FEV_1_/FVC < 0.7, FEV_1_ ≤ 60% predicted, and lung hyperinflation (TLC and/or FRC above the upper limit of normal [ULN] or > 120% predicted, measured by plethysmography). In addition, patients were symptomatic (CAT ≥ 10 at the screening and baseline visits), and had a history of ≥ 1 moderate or severe COPD exacerbation (see the Additional file 1 for definitions) in the previous 12 months. All patients had been on a stable twice-daily regimen of a non-extrafine ICS/LABA DPI for ≥ 8 weeks prior to screening. The main exclusion criteria were a current diagnosis of asthma or a number of other clinical conditions that could impact efficacy and/or safety evaluations or put the patient’s general wellbeing at risk. These conditions included pulmonary disorders other than COPD and acute COPD exacerbations within 4 weeks prior to inclusion. The full lists of inclusion and exclusion criteria are in the Additional file 1.

All patients provided written informed consent prior to any study-related procedure. The study was approved by independent ethics committees at each institution, and was performed in accordance with the principles of the Declaration of Helsinki and Good Clinical Practice. The study was registered at ClinicalTrials.gov (NCT04876677).

### Outcomes

The primary objective was to assess the effect on airway geometry and lung function of switching from high-dose FP/SLM DPI to medium-dose BDP/FF/G DPI. The primary endpoints were percentage changes in siV_aw_ and siR_aw_ from baseline (pre-dose at Visit 2) to pre-dose at Visit 3 (i.e., the effects of chronic dosing), assessed at TLC. The siV_aw_ and siR_aw_ were determined in central and distal lung regions, with the boundary between these regions defined by the third airway bifurcation. The siV_aw_ comparisons were made on ‘untrimmed’ airways such that all visible generations in a given scan (from Visit 2 or 3 respectively) were used in order to capture all available volume information. In contrast, siR_aw_ was evaluated using ‘trimmed’ data, meaning that only airway generations visible in both scans (i.e., the same airways) were used. The number of airway generations observed on MDCT within a given patient can vary between scans, and the use of trimmed data thus ensures that any differences between scans are due only to changes in airway calibre and not to differences in the number of airways used to make the CFD calculations.

Secondary endpoints included:

- The percentage changes from baseline (pre-dose at Visit 2) to post-dose at Visit 2, and from pre-dose to post-dose at Visit 3 in siV_aw_ and siR_aw_ at TLC (i.e., the effects of acute dosing at V2 and V3, respectively), assessed in the central and distal lung regions.
- The percentage changes from baseline to pre-dose at Visit 3, from baseline to post-dose at Visit 2, and from pre-dose to post-dose at Visit 3 in:
  - siV_aw_ and siR_aw_ at FRC, assessed in the central and distal lung regions;
  - volume fraction of total pulmonary vascular volume contained in blood vessels < 5 mm^2^ (BV5Pr), 5–10 mm^2^ (BV5–10Pr), and > 10 mm^2^ (BV10Pr), assessed in the total lung region;
  - image-based lobar volume at TLC and FRC, assessed in the total lung region;
  - and air trapping assessed via FRI at FRC, assessed in the total lung region.

In addition, pre-dose spirometry (FEV_1_ and FVC), body plethysmography (FRC, residual volume [RV], and TLC), and CAT total score were assessed at Visits 2 and 3.

### Sample size and statistical methods

As this was an exploratory study, there was no formal sample size calculation. It was considered sufficient for 25 patients to complete the study, with a planned enrolment of 30.

Data for the primary endpoints were log-transformed and analysed using a mixed model for repeated measures (MMRM) that included the log of baseline values (for untrimmed parameters) at TLC and visit number as covariates, and the interaction between visit and log of baseline values (for untrimmed parameters). The MDCT/FRI secondary efficacy endpoints were analysed using a similar MMRM to the primary endpoint, with the appropriate selection of the baseline (i.e., at TLC or FRC depending on the lung level selected). Spirometry, plethysmography and CAT endpoints were analysed using a paired t-test.

The safety set, used for the safety analyses, included all patients who received at least one dose of the study drug. Efficacy analyses were performed on the per protocol set, which excluded patients who did not have a post-baseline FRI evaluation or who had important protocol deviations impacting the FRI evaluation.

## Results

### Participants

The study was conducted between 18th May 2021 and 3rd January 2022 in two investigational sites, one each in Belgium and Hungary. Of 45 screened patients, 14 did not meet the inclusion/exclusion criteria, five withdrew consent, and one had an adverse event, with the remaining 25 recruited, all of whom completed the study. All recruited patients were included in the safety analysis; two patients were excluded from the per-protocol set as they had respiratory disorders other than COPD as determined by CT scan image analysis. Table 1 shows the baseline characteristics of the recruited patients. All patients had some degree of emphysema on CT evaluation by the sponsor’s medical expert.

**Table 1:** Baseline demographics and disease characteristics

Parameter	Value (N = 25)
Age, years	65.0 (7.7) [45; 79]
Sex at birth, male	16 (64.0%)
Race, white	25 (100%)
Body-mass index, kg/m^2^	27.7 (5.1) [17.0; 39.9]
Smoking status at screening	
Ex-smoker	10 (40.0%)
Current smoker	15 (60.0%)
Time since COPD diagnosis, years	10.1 (5.63)
Moderate/severe COPD exacerbations in the prior 12 months	1.7 (0.9)
Post-bronchodilator FEV_1_	
Absolute value, L	1.20 (0.46) [0.46; 2.31]
Percent predicted	43.5 (12.2) [20.3; 59.4]
Post-bronchodilator FVC	
Absolute value, L	2.35 (0.84) [1.12; 4.71]
Percent predicted	65.7 (15.2) [41.7; 91.3]
Post-bronchodilator FEV_1_/FVC	0.513 (0.092) [0.36; 0.69]
Functional residual capacity, percent predicted	157.7 (24.6) [122.0, 219.1]
Residual volume, percent predicted	190.9 (50.4) [91.2, 325.1]
Total lung capacity, percent predicted	118.6 (18.1) [84.7, 155.8]
CAT score	19.7 (3.9) [12; 28]

Data are mean (SD) [range] or number (%). FEV_1_: forced expiratory volume in 1 s; FVC: forced vital capacity; CAT: COPD Assessment Test

### Outcomes

**Fig. 2:**
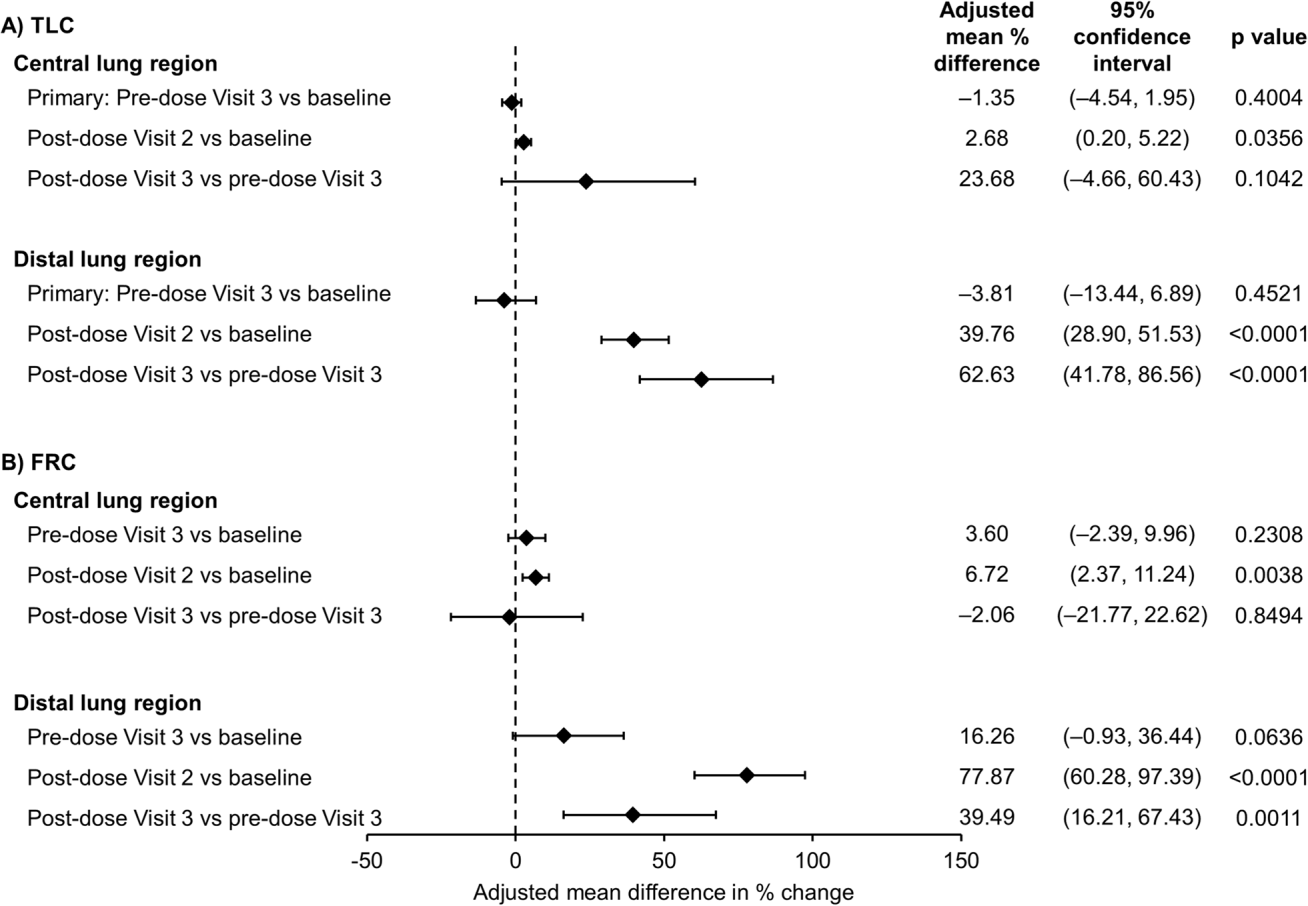
Airway volume (siV_aw_) at **A** TLC and **B** FRC (per protocol set). Data are from 23 patients. siV_aw_: specific image-based airway volume; TLC: total lung capacity; FRC: functional residual capacity. Baseline data were assessed pre-dose at Visit 2

**Fig. 3:**
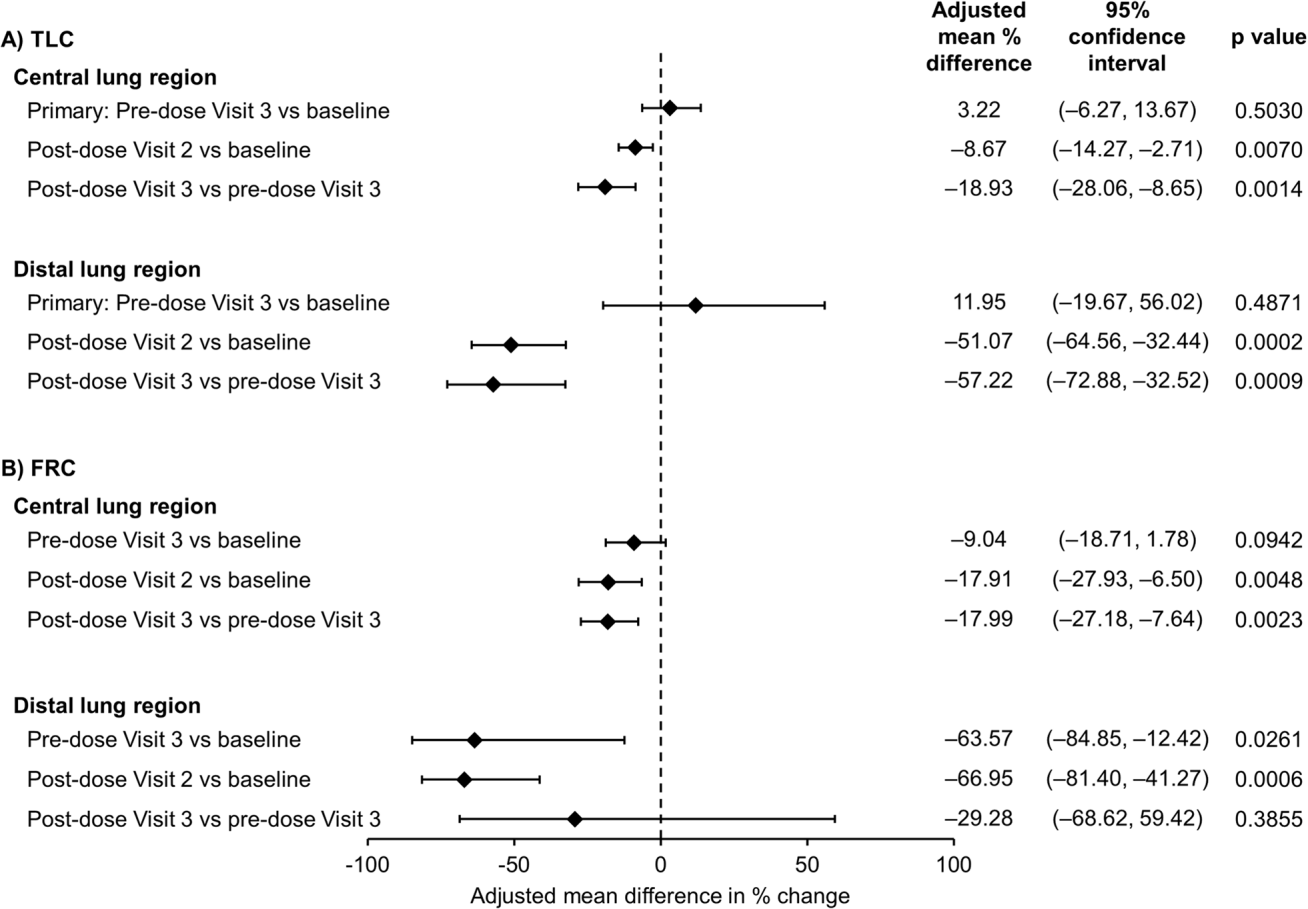
Airway resistance (siR_aw_) at TLC and FRC (per protocol set). Data are from 23 patients. siR_aw_: specific image-based airway resistance; TLC: total lung capacity; FRC: functional residual capacity. Baseline data were assessed pre-dose at Visit 2

After 6 weeks of treatment with BDP/FF/G there were no significant changes from baseline in pre-dose siV_aw_ or siR_aw_ assessed at TLC [the primary endpoints (Figs. 2 and 3)]. However, at FRC in the distal lung region there was a trend toward an increase from baseline after 6 weeks in pre-dose siV_aw_, with a statistically significant decrease in siR_aw_. In addition, there were significant acute effects on siV_aw_, with statistically significant increases in the distal airways from pre- to post-dose at TLC and FRC on both visits, and in the central airways on Visit 2 (Fig. 2). Similarly, there were significant acute effects on siR_aw_, with statistically significant decreases in the distal airways from pre- to post-dose at TLC on both visits and at FRC at Visit 2 (with a numerical decrease at Visit 3), and in the central airways on both visits (Fig. 3).

**Fig. 4:**
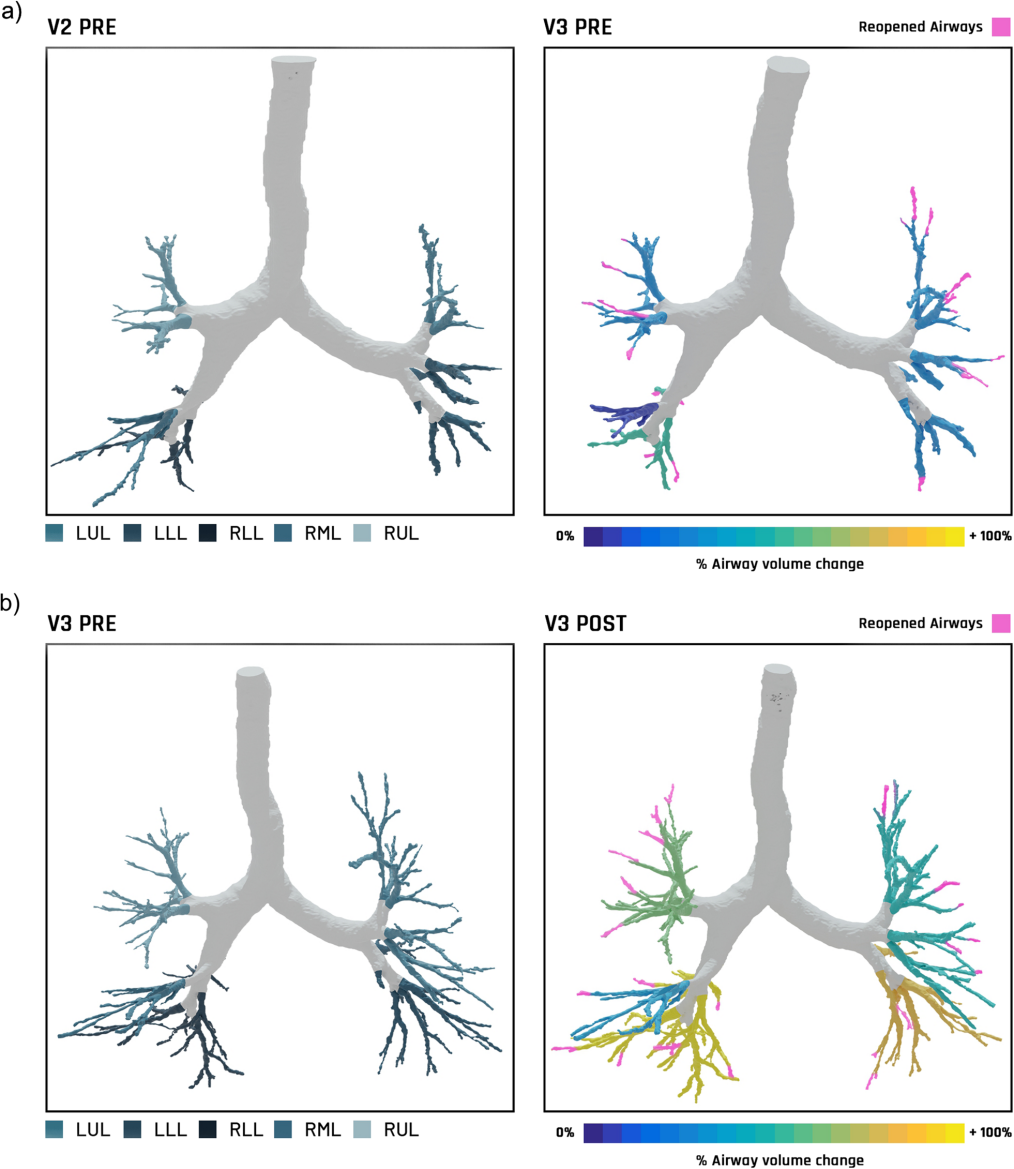
Example Functional Respiratory Imaging lung scans. The left-hand figures are pre-dose, with the lung regions colour coded. The right-hand figures illustrate the percent airway volume change resulting from administration of BDP/FF/G, demonstrating the effects of **a** chronic dosing (i.e., from pre-dose at Visit 2 [baseline] to pre-dose at Visit 3), taken at functional residual capacity, and **b** acute dosing (from pre-dose to post-dose at Visit 3), taken at total lung capacity. The airways coloured pink in each right-hand image are airways that were not visible in the matching left-hand image. BDP/FF/G: beclometasone dipropionate/formoterol fumarate dihydrate/glycopyrronium; V2 PRE: Visit 2 pre-dose (i.e., baseline); V3 PRE: Visit 3 (i.e., after 6 weeks) pre-dose; LUL: left upper lobe; LLL: left lower lobe; RLL: right lower lobe; RML: right middle lobe; RUL: right upper lobe; V3 POST: Visit 3, assessed following administration of BDP/FF/G

These results are illustrated in the examples of Functional Respiratory Imaging lung scans in Fig. 4. Following chronic (i.e., after 6 weeks) and acute dosing, BDP/FF/G produced increases in distal airway volumes, comprising both increases in the calibre of airways that were visible at baseline, and the appearance of airways that were previously too small to be seen (coloured pink in the right-hand scans). Both of these effects had a degree of regional heterogeneity in effect size.

For the secondary MDCT/FRI endpoints, following acute dosing there were trends toward an increased volume fraction of the smallest blood vessels (BV5Pr) and decreased volume fraction of the largest vessels (BV10Pr), with decreases in lobar volume at TLC and FRC following the first dose, although differences were not consistently statistically significant (Additional file 1: Table S1). These were accompanied by significant decreases in air trapping at FRC (Additional file 1: Table S1).

There were no significant changes in the spirometry or plethysmography parameters, with a change from baseline in pre-dose FEV_1_ at Visit 3 of 62.2 mL (95% CI − 5.3, 129.6 mL; p = 0.0690) (Additional file 1: Table S2). However, there was a statistically significant improvement from baseline (i.e., reduction) in CAT total score at Visit 3 (mean change from baseline − 3.30 [95% CI − 4.62; − 1.98]; p < 0.0001).

### Safety

Eleven (44.0%) patients experienced adverse events. None were considered serious or related to the study, and none resulted in study discontinuation. The only preferred terms that occurred in more than one patient were headache (three patients [12.0%]), mild in intensity, and pleural calcification (two patients [8.0%]), noted incidentally on CT scan. There were no clinically significant changes in any of the vital signs, 12-lead electrocardiograms, or physical examination assessments.

## Discussion

The primary endpoints of this study evaluated the effect of chronic dosing (i.e., the change in pre-dose values after 6 weeks) with medium-dose BDP/FF/G in terms of airway volume and resistance at TLC after switching from high-dose FP/SLM (Figs. 2 and 3). There were no marked differences in any of these parameters, or in the spirometry or plethysmography endpoints (Additional file 1: Table S2), although the change from baseline in pre-dose FEV_1_ after 6 weeks (62 mL) was consistent with the 47 mL increase after 4 weeks in the BDP/FF/G arm in the TRI-D study in which patients had a 2-week ICS/LABA run-in [4]. Overall, this indicates that reducing the ICS dose and adding the LAMA component results in a treatment that is at least as effective as high-dose FP/SLM. At FRC, however, there were improvements in the distal lung following 6 weeks of treatment that approached significance for airway volume [i.e., an increase (Fig. 2)], and reached significance for airway resistance [i.e., a decrease (Fig. 3)]. Furthermore, there were acute improvements in airway volume and resistance at both TLC and FRC, especially in the distal lung, with similar results at Visit 2 (first dose) and Visit 3 (after 6 weeks) at TLC. This indicates that there is no loss of efficacy following repeat dosing of BDP/FF/G. However, the magnitudes of the acute changes at FRC were somewhat less at Visit 3 than Visit 2. A possible explanation for this is that the improvements in airway volume and resistance after 6 weeks of maintenance treatment with BDP/FF/G arm diminished the potential for additional improvements at Visit 3.

The improvements in volume and resistance in the distal airway region area are noteworthy as this region most closely reflects the behaviour of the small airways, which are the major site of airflow obstruction in all severities of COPD due to a loss of terminal bronchioles [7, 8]. In addition, gas trapping due to occlusion of small airways correlates with a number of clinical outcomes in patients with COPD, including decreased 6 min walk distance, increased exacerbation frequency, and worsened health status and dyspnoea [9]. Furthermore, in a previous study that used impulse oscillometry to evaluate distal airway involvement in 202 patients with COPD, increasing small airways resistance correlated with worsening health status (assessed using CAT total score) [10]. The results of this study thus suggest that the administration of dual bronchodilation (LABA plus LAMA) in BDP/FF/G improves airway smooth muscle tone, especially in the distal airways, leading to an increase in airway calibre and a reduced propensity for airway collapse on exhalation (i.e., at FRC).

The different outcomes observed at FRC versus TLC may reflect the fact that the outward parenchymal tethering forces on the airways at TLC are large enough to make any therapeutic alterations in airway smooth muscle tone irrelevant in terms of their effects on airway calibre. These results would appear to contrast with those of van den Berge et al*.* who used FRI in patients with COPD to evaluate siV_aw_ and siR_aw_ at TLC in the whole lung [11]. They found significant improvements in siV_aw_ and siR_aw_ at TLC following 28 days administration in patients receiving budesonide/glycopyrrolate/FF or glycopyrrolate/FF compared to those receiving ipratropium. However, that study used a crossover design, rather than switching patients from ICS/LABA to inhaled triple therapy as in DARWiIN. Furthermore, in the prior study, follow-up assessments were only conducted 90 min post-dose, during which the effects of acute bronchodilation were ongoing. In contrast, in DARWiIN, the primary follow-up was at pre-dose, so only chronic rather than acute effects were reflected in the comparison. The significant acute changes observed at TLC at both Visit 2 and Visit 3 (Figs. 2 and 3) therefore suggest that, had a similar comparison been made in the prior study, the results of the two studies would have been similar.

Even though meaningful changes at TLC were not found in either pulmonary function or FRI airway indices, there was nevertheless a clinical improvement as indicated by the CAT score. This may have been the result of improved perfusion since, following acute dosing of BDP/FF/G, there were trends toward increased volume fraction of the smallest blood vessels and decreased volume fraction of the largest vessels (Additional file 1: Table S1). These changes are consistent with those that occur following administration of a pulmonary vasodilator [12], raising the possibility that treatment with the study drug reversed a degree of hypoxic vasoconstriction that may have been present, since chronic hypoxia is characteristic of some patients with COPD [13], particularly those with advanced disease. In addition, the acute decreases in lobar volume we observed at TLC and FRC, likely reflective of reduced gas trapping, may have contributed to improved clinical status despite not being reflected in the change from baseline to pre-dose at Visit 3. Improved perfusion and reduced gas trapping would both be expected to result in improved gas exchange.

The main limitations of the DARWiIN study are the relatively small sample size and the lack of a blinded comparator arm. Furthermore, the study was not designed to evaluate the potential safety and tolerability benefits of reducing the ICS dose, although many of the adverse effects of ICS are dose-related, most notably the incidence of pneumonia [14]—no occurrences of which were reported during the study. Indeed, BDP/FF/G was well tolerated in these patients and had a good overall safety profile, consistent with the results of larger, longer-term studies in patients with COPD [1–3].

## Conclusion

In patients with COPD who are symptomatic when receiving high-dose ICS/LABA, adding a LAMA while decreasing the ICS dose by switching to medium-dose extrafine BDP/FF/G improves indices of airway function, especially in the distal airways, and leads to marked improvements in health status. This strategy thus at least maintains, and possibly improves, efficacy while reducing the potential for dose-related adverse effects associated with ICS use.

### Supplementary Information

**Additional file 1.** Supplementary methods and results.

## Data Availability

Chiesi commits to sharing with qualified scientific and medical researchers, conducting legitimate research, the anonymised patient-level and study-level data, the clinical protocol and the full clinical study report of Chiesi Farmaceutici SpA-sponsored interventional clinical trials in patients for medicines and indications approved by the European Medicines Agency and/or the US Food and Drug Administration after 1st January 2015, following the approval of any received research proposal and the signature of a Data Sharing Agreement. Chiesi provides access to clinical trial information consistently with the principle of safeguarding commercially confidential information and patient privacy. Other information on Chiesi’s data sharing commitment, access and research request’s approval process are available in the Clinical Trial Transparency section of http://www.chiesi.com/en/research-and-development/.

## Abbreviations

BDP/FF/G: Beclometasone dipropionate/formoterol fumarate dihydrate/glycopyrronium
BV5Pr: Volume fraction of total pulmonary vascular volume contained in blood vessels < 5 mm^2^
BV5–10Pr: Volume fraction of total pulmonary vascular volume contained in blood vessels 5–10 mm^2^
BV10Pr: Volume fraction of total pulmonary vascular volume contained in blood vessels > 10 mm^2^
CAT: COPD Assessment Test
CFD: Computational fluid dynamics
COPD: Chronic obstructive pulmonary disease
CT: Computed tomography
DPI: Dry powder inhaler
FEV_1_: Forced expiratory volume in 1 s
FP/SLM: Fluticasone propionate/salmeterol
FRC: Functional residual capacity
FRI: Functional Respiratory Imaging
FVC: Forced vital capacity
ICS: Inhaled corticosteroid
LABA: Long-acting β_2_-agonist
MDCT: Multidetector computed tomography
MMRM: Mixed model for repeated measures
pMDI: Pressurised metered dose inhaler
RV: Residual volume
siR_aw_: Specific image-based airway resistance
siV_aw_: Specific image-based airway volume
TLC: Total lung capacity
ULN: Upper limit of normal
