# Supplementary material for: The effect of inhaled extrafine beclometasone dipropionate/formoterol fumarate/glycopyrronium bromide on distal and central airway indices, assessed using Functional Respiratory Imaging in COPD (DARWiIN)
Source: Respir Res. 2023 Oct 6;24:244. doi: 10.1186/s12931-023-02549-5 (PMC10559640; doi:10.1186/s12931-023-02549-5)
Supplement: Supplementary file 1 — Additional file 1. Supplementary methods and results. [file 12931_2023_2549_MOESM1_ESM.pdf]

The effect of inhaled extrafine beclometasone dipropionate/formoterol fumarate/glycopyrronium bromide on distal and central airway indices, assessed using Functional Respiratory Imaging in COPD (DARWiIN)

Authors

Gwen S Skloot, Alessandro Guasconi, Benjamin R Lavon, George Georges, Wilfried De Backer, Dmitry Galkin, Mauro Cortellini, Ilaria Panni, Jason HT Bates

Supplementary materials

Methods

Inclusion criteria

1. Patient's signed ICF obtained prior to any study-related procedure;
2. Male or female ≥40 years of age;
3. Current smokers or ex-smokers of at least 10 pack-years, calculated as (number of cigarettes/day * number of years)/20 (e-cigarettes smoking could not be used to calculate pack-year history);
4. Established diagnosis of COPD according to the 2020 GOLD Report, prior to the V1;
5. Post-bronchodilator forced expiratory volume in 1 sec/forced vital capacity (FEV_1/FVC) <0.7 and FEV_1 ≤60% of predicted at V1 (Note: if the criterion was not met at screening, the measure could be repeated once before run-in Day 1);
6. On a stable dose of any non-extrafine ICS/LABA DPI twice daily regimen for at least 8 weeks before screening;
7. Presence of lung hyperinflation based on the increase of TLC exceeding either the upper limit of normal (ULN) or 120% of predicted, and/or a plethysmographic FRC exceeding either ULN or 120% of predicted;
8. Symptomatic patients with COPD Assessment Test (CAT) score ≥10 at V1 and V2;
9. Documented history of ≥1 moderate or severe COPD exacerbation in the previous 12 months prior to V1;
10. Had a cooperative attitude and the ability to be trained and use correctly the DPIs;
11. Had a cooperative attitude and the ability to perform the required outcomes measurements (e.g., spirometry manoeuvres in sitting and supine position) and the ability to understand the risks involved;
12. Women of childbearing potential (WOCBP) fulfilling one of the following criteria: a. WOCBP with fertile male partners: they and/or their partner had to be willing to use a highly effective birth control method from the signature of the informed consent and until the follow-up visit or b. WOCBP with non-fertile male partners (contraception was not required in this case).
13. Female patients of non-childbearing potential defined as physiologically incapable of becoming pregnant (i.e., post-menopausal or permanently sterile; e.g., amenorrhea

for ≥12 consecutive months without alternative medical cause). Permanent sterilisation methods included hysterectomy, bilateral salpingectomy, and bilateral oophorectomy. If indicated, as per Investigator's request, post-menopausal status could be confirmed by follicle-stimulating hormone (FSH) levels (according to local laboratory ranges).

Exclusion criteria

1. Pregnant or lactating woman;
2. Exacerbations defined as a sustained and acute deterioration of patient's symptoms and signs (dyspnoea, cough and/or sputum production/purulence) that were either moderate, i.e., required treatment with systemic (oral/intravenous [IV]/intramuscular [IM]) corticosteroids and/or antibiotics, or severe, i.e., required hospitalisation, if their associated treatment/hospitalisation occurred within the 30 days before V1 (or 4 weeks in case the event was treated with just systemic corticosteroids) or if the event was recorded during the run-in period;
3. A current asthma diagnosis;
4. Respiratory disorders other than COPD: patients with known respiratory disorders other than COPD that in the Investigator's opinion could affect efficacy and safety evaluation or place the patient at risk. This could include but was not limited to known α1-anti-trypsine deficiency, active tuberculosis, bronchiectasis, sarcoidosis, lung fibrosis, pulmonary hypertension, and interstitial lung disease;
5. Cardiovascular diseases: patients who had known clinically significant cardiovascular conditions such as but not limited to: unstable or acute ischaemic heart disease within one year prior to study entry, New York Heart Association (NYHA) class IV heart failure, history of atrial fibrillation, history of sustained, and non-sustained cardiac arrhythmias diagnosed within 6 months prior to study entry and not controlled with therapy according to the Investigator's opinion;
6. Evidence or history of other concurrent disease such as but not limited to hyperthyroidism, diabetes mellitus, or other endocrine disease; haematological disease; autoimmune disorders (e.g,. rheumatoid arthritis); significant renal impairment; significant neurological disease or other disease or condition that might, in the judgement of the Investigator, place the patient at undue risk or potentially compromise the results or interpretation of the study;

7. Medical history or current diagnosis of narrow-angle glaucoma, clinically relevant prostatic hypertrophy, or bladder neck obstruction that in the opinion of the Investigator could have prevented use of anticholinergic agents;

8. History of lung transplant or lung reduction surgery;

9. Electrocardiogram (ECG) criteria: any clinically significant abnormal 12-lead ECG that in the Investigator’s opinion could affect efficacy or safety evaluation or place the patients at risk. Male patients with a QTcF >450 ms and female patients with a QTcF >470 ms at V1 were not eligible;

10. Laboratory abnormalities: patients with clinically significant laboratory abnormalities indicating a significant or unstable concomitant disease that could, in the judgement of the Investigator, place the patient at undue risk or potentially compromise the results or interpretation of the study;

11. Alcohol/drug abuse: patients with a known or suspected history of alcohol and/or substance/drug abuse within 12 months prior to screening; or had a positive drug test at screening or V2;

12. Participation in investigational study: patients who had received any investigational drug within the 30 days or a more appropriate time as determined by the Investigator (e.g., approximately five half-lives of the investigational drug, whatever was longer);

13. Contra-indications to investigational medicinal products (IMPs), based on Investigator judgement;

14. Hypersensitivity: history of hypersensitivity to any of the study medications components or a history of other allergy that in the opinion of the Investigator contraindicated the patient’s participation;

15. Patients mentally or legally incapacitated or patients accommodated in an establishment as a result of an official or judicial order;

16. Documented Coronavirus disease 2019 (COVID-19) diagnosis or its complications which had not resolved within 14 days prior to screening;

17. Positive molecular COVID-19 test within the last 72 h before the remaining of screening activities.

Multidetector computed tomography/Functional Respiratory Imaging endpoints

Airway volume

The airways can be segmented up to the point where no distinction can be made between the intraluminal and alveolar air. This is where the airway diameter is around 1–2 mm, typically around the 7th-10th bifurcation, depending mainly on the disease state of the individual patient. From the resulting model, central and distal iV_{aw} can be assessed at individual airways or in different regions. The distal airway volume is defined as the segmented airway volume starting from the 3rd bifurcation. Visible airway generations can be different in the different visit scans. Trimmed airway volume indicates the volume of the generations of airways that are visible in the scans of all study visit days.

The siV_{aw} as an FRI parameter is derived from the iV_{aw}. The specificity is calculated by dividing the airway volume with the lung volume. This way, the airway volumes are normalised across patients and become specific. Indeed, if two patients have the same airway volume, but have different lung volumes and thus different lung sizes, the patient with the larger lungs will have relatively smaller airway volumes. By dividing the airway volumes with the lung volumes, a comparison between lungs of varied sizes is possible. Also, if a treatment changes both lung and airway volumes (e.g., most treatments in COPD increase airway volumes whilst they decrease lung volume) the specific airway volumes often give an enhanced signal.

Airway resistance

The iR_{aw} is determined using computational fluid dynamics (CFD). During the CFD calculations, the outflow to each lobe is adjusted iteratively for each patient to match the internal flow rate distributions obtained from the segmentation of the CT scans. As a result, iR_{aw} accounts for the patient-specific internal airflow distribution which might be greatly altered by the lung disease. Hence, the airflow distribution in the CFD calculations reflects

the airflow distribution as derived from the expansion of lung lobes from FRC to TLC. The iR_{aw} is defined as the total pressure drop over an airway, divided by the flow rate through that airway.

The CT images at total lung capacity capture the first 7–10 generations. The airways beyond the 10th generation cannot be directly visualised from the CT images but their characteristics can be inferred by including lobe expansion and hence the patient-specific internal airflow distribution in the CFD-based resistance calculation.

By segmenting the lobes at FRC and TLC for each patient, the patient-specific airflow distribution can be established by assessing lobar and volume expansion. The siR_{aw} as an FRI parameter is derived from the iR_{aw}. The specificity is calculated by multiplying the airway resistances with the lung resistances. This way, the airway resistances are normalised across patients and become specific, permitting comparison between patients.

Perfusion mapping

The volume of segmented pulmonary vessels are divided into three categories according to cross-sectional area, with BV5, BV5–10, and BV10 referring to vessels <5 mm^2, 5–10 mm^2, and >10 mm^2 in cross sectional area, respectively. These volumes are the combined volumes of the pulmonary arteries and veins, and because of variations in blood vessel volume based on overall body size, these measures are often expressed using the ratio to total blood volume (TBV). This way each of the measures expresses the fraction of blood vessel calibre present in each category. Lower values in small vasculature (<5 mm^2) may reflect CT imaging evidence of vascular pruning, i.e., a smaller proportion of the blood vessel volume comprised of small peripheral pulmonary blood vessels. The BVX data is the absolute value, expressed in mL. BVXPR data is expressed as % of all regional blood vessel volume.

Lobar volume

Lobar volume (iV_{lobe}) is an FRI-based ventilation parameter obtained by identifying and grouping voxels that represent the air in the lungs.

Air trapping

FRI-based air trapping is defined as all the intrapulmonary voxels with Hounsfield Units between –1024 and –850 using the expiratory scans at FRC.

Results

Supplementary Table 1. Secondary multidetector computed tomography/functional respiratory imaging endpoints (per protocol set).

Parameter	Baseline (pre-dose Visit 2), mean (SD)	Change, adjusted mean % difference (95% confidence interval); p value		
		Pre-dose Visit 3 vs baseline	Post-dose Visit 2 vs baseline	Post-dose Visit 3 vs pre-dose Visit 3
Volume fraction of total pulmonary vascular volume contained in blood vessels, total lung region, %				
BV5Pr	28.94 (11.19)	0.43 (–2.49, 3.43); 0.7670	6.39 (0.52, 12.59); 0.0338	16.39 (–37.66, 117.28); 0.6184
BV5–10Pr	26.35 (6.37)	–0.54 (–3.75, 2.77); 0.7342	1.39 (–0.99, 3.83); 0.2400	11.78 (–17.01, 50.56); 0.4453
BV10Pr	20.06 (4.52)	–0.14 (–4.19, 4.08); 0.9435	–1.37 (–4.80, 2.19); 0.4275	–13.61 (–38.00, 20.37); 0.3694
Image-based lobar volume, total lung region, log, L				
At TLC	6.39 (1.44)	0.30 (–2.33, 2.99); 0.8190	–1.92 (–4.91, 1.15); 0.2048	–2.55 (–20.14, 18.92); 0.7899
At FRC	4.41 (1.10)	1.75 (–2.55, 6.24); 0.4121	–3.22 (–6.33, –0.01); 0.0495	6.19 (–17.49, 36.67); 0.6256
Air trapping at FRC, total lung region, %	52.38 (20.51)	2.76 (–5.09, 11.26); 0.4843	–8.38 (–15.23, –0.97); 0.0292	–50.98 (–67.23, –26.67); 0.0014

Data are from 23 patients. BV5Pr, BV5–10Pr and BV10Pr, volume fraction of total pulmonary vascular volume contained in blood vessels with cross-sectional area <5 mm^2, 5–10 mm^2, and >10 mm^2, respectively, with baseline values being the volume of blood vessels in each category; TLC, total lung capacity; FRC, functional residual capacity.

Supplementary Table 2. Spirometry and body plethysmography (per protocol set).

**Parameter**	**Baseline (pre-dose Visit 2), mean (SD)**	**Pre-dose Visit 3, change from baseline, mean (95% CI); p value**
Forced expiratory volume in 1 sec, L	1.187 (0.401)	0.0622 (–0.0053; 0.1296); 0.0690
Forced vital capacity, L	2.350 (0.726)	0.1030 (–0.0339; 0.2400); 0.1330
Functional residual capacity, L	4.741 (0.912)	0.1248 (–0.2243; 0.4738); 0.4663
Residual volume, L	4.058 (0.901)	–0.0335 (–0.3185; 0.2516); 0.8098
Total lung capacity, L	6.708 (1.566)	–0.0561 (–0.3151; 0.2029); 0.6577

Data are from 23 patients.
